# Compound danshen dripping pills vs. nitrates for stable angina pectoris: a systematic review and meta-analysis

**DOI:** 10.3389/fcvm.2023.1168730

**Published:** 2023-05-22

**Authors:** Mengying Zhang, Wenjia Wang, He Sun, Jingbo Zhai, Yunhui Hu

**Affiliations:** ^1^ Tasly Pharmaceutical Group Co., Ltd., Tianjin, China; ^2^ The State Key Laboratory of Core Technology in Innovative Chinese Medicine, Tasly Academy, Tasly Holding Group Co., Ltd., Tianjin, China; ^3^ School of Public Health, Tianjin University of Traditional Chinese Medicine, Tianjin, China

**Keywords:** compound danshen dripping pills (CDDP), nitrates, stable angina pectoris (SAP), systematic review, meta-analysis

## Abstract

**Background:**

Long-term use of nitrates for treating stable angina pectoris (SAP) may lead to patients' tolerance to nitrates. As a traditional Chinese medicine, Compound danshen dropping pills (CDDP) is beneficial for patients with SAP. This study aimed to critically assess the efficacy and safety of CDDP vs. nitrates for SAP.

**Methods:**

PubMed, Embase, Web of Science, Cochrane library, CNKI, Wanfang Digital Periodicals, and Chinese Science and Technology Periodicals database were searched from inception to April 2023. Randomized controlled trials (RCTs) comparing CDDP with nitrates for SAP were included. The meta-analysis was conducted to estimate the pooled effect.

**Results:**

Twenty-nine studies were included for the statistical analysis. The meta-analyses with the random-effect model indicated that CDDP could significantly increase the effective rate in symptom improvement compared with nitrates (Pooled 9 RCTs, OR = 1.95, 95% CI: 1.25–3.05, *P* = 0.003, duration of 4 weeks; Pooled 4 RCTs, OR = 3.45, 95% CI: 1.84–6.48, *P* = 0.0001, duration of 6 weeks; Pooled 13 RCTs, OR = 4.02, 95% CI: 2.14–7.57, *P* < 0.0001, duration of 8 weeks). The meta-analyses with the random-effect model indicated that CDDP could significantly increase the effective rate in electrocardiogram improvement compared with nitrates (Pooled 5 RCTs, OR = 1.60, 95% CI: 1.02–2.52, *P* = 0.04, duration of 4 weeks; Pooled 3 RCTs, OR = 2.47, 95% CI: 1.60–3.82, *P* < 0.0001, duration of 6 weeks; Pooled 11 RCTs, OR = 3.43, 95% CI: 2.68–4.38, *P* < 0.00001, duration of 8 weeks). The incidence of adverse drug reactions in the CDDP group was lower than that in the nitrates group (Pooled 23 RCTs, OR = 0.15, 95% CI: 0.1–0.21, *P* < 0.00001). The results of the meta-analyses with fixed-effect model were similar with above results. The levels of the evidence ranged from very low to low.

**Conclusion:**

The present study suggests that CDDP with the duration of at least 4 weeks can be considered as an alternative to nitrates for treating SAP. However, more high-quality RCTs are still needed to confirm these findings.

**Systematic Review Registration:**

https://www.crd.york.ac.uk/prospero/display_record.php?ID=CRD42022352888, identifier [CRD42022352888].

## 1. Introduction

Stable angina pectoris (SAP) is one of the most common manifestations of coronary artery disease (CAD) (1). A survey reported that the prevalence of chronic SAP was 7.7% in Iran (2). A prospective study showed that 20% of patients with CAD had symptoms related with angina pectoris, which may be associated with an increased risk of adverse cardiovascular outcomes (3). A cohort study found that the high frequency of angina pectoris was associated with the high incidence of secondary cardiovascular events and death in patients with stable coronary heart disease (4).

At present, the goals of treatments for SAP mainly focus on the reduction in cardiovascular adverse events and the improvement in the quality of life (5). There are many antianginal therapies, such as nitrates, beta-blockers, calcium channel blockers and ranolazine (5). Nitrates as standard antianginal treatments have been used in the management of angina pectoris for many years (6). A systematic review found that nitrates could significantly reduce the angina attacks and improve the exercise duration (6). However, some adverse events associated with nitrates are reported, such as headache and dizziness (6). Moreover, long-term use may lead to patients' tolerance to nitrates and the reduction of protection duration (7).

Traditional Chinese medicine (TCM) is beneficial for the management of angina pectoris. A systematic review showed that TCM could reduce the frequency of angina pectoris, shorten the duration of angina attack and improve the quality of life (8). Compound danshen dropping pills (CDDP) is a TCM formula, and is composed of Salvia miltiorrhiza Bunge, Panax notoginseng (Burkill) F.H. Chen and Dryobalanops aromatica C.F. Gaertn. TCM network pharmacology is a new approach to understanding herb formula (9, 10). A study based on the network pharmacology and molecular docking found that the mechanisms of CDDP for treating SAP might be associated with the regulation of inflammatory response, apoptosis signal pathway, etc. (11). Some clinical trials comparing CDDP with nitrates for SAP have been published in recent years. However, the relative advantages and disadvantages of CDDP compared with nitrates for SAP were not systematically evaluated. Therefore, we conducted this systematic review to critically assess the efficacy and safety of CDDP vs. nitrates for SAP.

## 2. Methods

This study was registered on PROSPERO (No. CRD42022352888) available from: https://www.crd.york.ac.uk/prospero/display_record.php?ID=CRD42022352888. It followed the Preferred Reporting Items for Systematic reviews and Meta-Analyses (PRISMA) statement (12).

## 2.1. Inclusion and exclusion criteria

### 2.1.1. Type of included studies

Randomized controlled trials (RCTs) were considered for inclusion, regardless of publication date and language. Quasi-RCTs, cross-over trials, on-going trials, abstracts and letters were excluded.

### 2.1.2. Patients

Patients diagnosed with SAP were included. Age, gender, race and nationality were unrestricted. SAP should be assessed according to the internationally recognized criteria or guidelines, such as National Institute for Health and Care Excellence or European Society of Cardiology guidelines (13).

### 2.1.3. Interventions

CDDP was used to treat SAP in the experimental group. Nitrates were used for the management of SAP in the control group. According to the instruction of CDDP, patients should take CDDP ten pills a time, and three times a day for at least 4 weeks. The dosage, frequency and course of nitrates were unlimited. The types of nitrates such as isosorbide dinitrate and isosorbide nitrate were also unrestricted. Other specific interventions for treating SAP were inhibited in the two groups. Patients with SAP may be accompanied with other diseases, such as coronary heart disease. Conventional therapies for the management of these accompanied diseases were unlimited.

### 2.1.4. Outcomes

The primary outcomes were the effective rate and markedly effective rate in symptom improvement (14). The secondary outcomes included the effective rate and markedly effective rate in electrocardiogram (ECG) improvement, and adverse drug reactions. The patients were labeled as “effective” in symptom improvement when the frequency of angina attacks and nitroglycerin consumption were reduced by more than 50%, and “markedly effective” in symptom improvement when symptoms associated with SAP were disappeared, the frequency of angina attacks or nitroglycerin consumption was reduced by more than 80% or 90% after treatment. The patients were labeled as “effective” in ECG improvement when S-T segment was elevated by 0.5 mm, however, not recovered completely after treatment, or defined by guidelines. The patients were labeled as “markedly effective” in ECG improvement when resting electrocardiogram returned to normal level after treatment, or defined by guidelines. The effective rate is equal to the number of patients labeled as “effective” or “markedly effective” divided by the total number of patients in one group. The markedly effective rate is equal to the number of patients labeled as “markedly effective” divided by the total number of patients in one group.

#### 2.2. Search strategy

Two reviewers (JZ and MZ) independently searched PubMed, Embase, Web of Science, Cochrane library, China National Knowledge Infrastructure, Wanfang Digital Periodicals, and Chinese Science and Technology Periodicals database from inception to April 2023. The search terms included (“compound danshen dripping pills” OR “fufang danshen diwan” OR “Cardiotonic Pills” OR “dantonic” OR T89) AND (nitrate OR “isosorbide dinitrate” OR “isosorbide nitrate”) AND (“stable angina” OR “angina” OR “angina pectoris” OR “stenocardia” OR “angor pectoris”). Moreover, ClinicalTrials.gov, World Health Organization International Clinical trials Registry platform, and Chinese Clinical Trial Registry were also be searched to identify potentially grey literatures. The detailed search strategies are attainable in Supplementary Material.

#### 2.3. Study selection

The studies collected from the comprehensive literature search were imported into EndNote X9 software to remove duplicates. Then, two reviewers (JZ and MZ) independently checked titles and abstracts to delete obviously ineligible studies according to the inclusion and exclusion criteria. They checked the full texts of the remaining studies to further identify eligible studies. Disagreements were handled in consultation with a third reviewer (YH). A PRISMA flow diagram was used to describe the screening process.

#### 2.4. Data extraction

The important data were extracted and imported into Microsoft Excel 2013 by two authors (JZ and MZ) independently. The information included characteristic of included studies (first author, publication year, country, sample size, design), patients (age, gender, race, nationality), interventions (type, dosage, frequency, duration), and outcomes (primary and secondary outcomes). The information on risk of bias assessment (randomization, allocation, blinding, loss to follow-up) were also extracted synchronously.

#### 2.5. Assessment of risk of bias

The risk of bias for included RCTs was assessed by the Cochrane “risk of bias” tool. The selection bias, performance bias, detection bias, attrition bias, and reporting bias for each included RCT was classified to low, high or unclear level. Two reviewers (JZ and MZ) were independently assessed the risk of bias. Any disagreement was resolved by a third author (YH).

#### 2.6. Statistical analysis

Odds ratio (OR) with 95% confidence intervals (CIs) was used to estimate the effect size for the dichotomous variables. The meta-analysis with the random-effect model as the primary analysis was conducted by Review Manager 5.4.1 software. Then, the meta-analysis with the fixed-effect model was conducted as the sensitivity analysis. *P* < 0.05 indicated a statistically significant difference between the two groups. Subgroup analyses were conducted based on the duration. The publication bias was assessed by the funnel plot. The level of evidence for each outcome was assessed by the Grading of Recommendations Assessment, Development, and Evaluation (GRADE) system.

## 3. Results

### 3.1. Literature search

**Figure 1:**
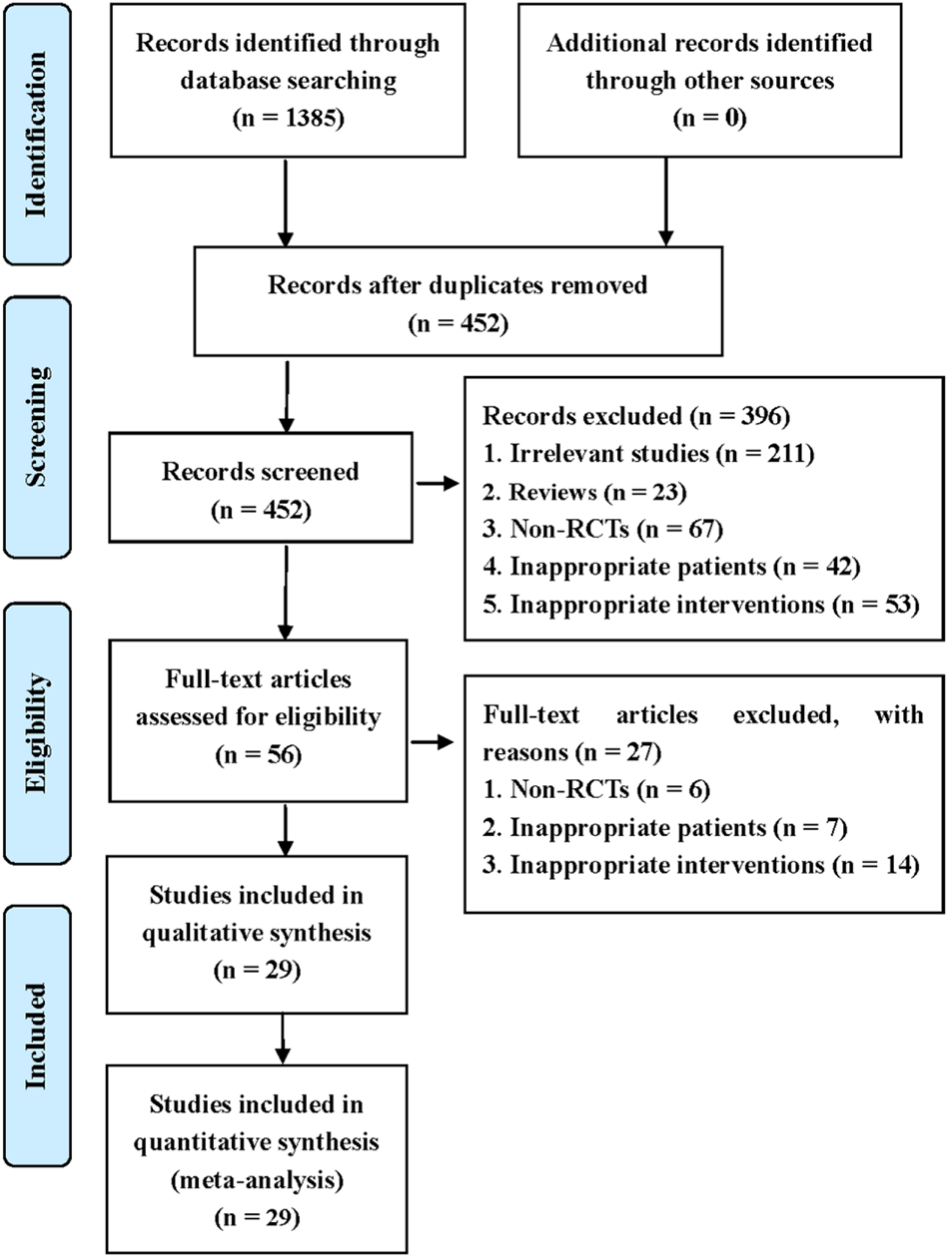
Flow diagram of study retrieval and selection.

This systematic review identified 1,385 potentially eligible studies via the extensive search. Nine hundred and thirty-three duplicate studies were removed using EndNote software. Then, 396 irrelevant studies were deleted by checking the titles and abstracts. After checking full-texts, 29 studies were finally included for the statistical analysis. The process of screening eligible studies is described in Figure 1 (15–43).

### 3.2. Characteristics of included studies

The characteristics of the included studies are summarized in Table 1. They involved 3,185 patients and were published between 1997 and 2013. Sample size ranged from 25 to 120 in the experimental group and 25–114 in the control group. Isosorbide nitrate was used in 7 trials (15, 16, 19, 21, 23, 24, 42), and isosorbide dinitrate was used in other studies. There was the same dosage of CDDP or nitrates across the included studies. The duration of CDDP or nitrates ranged from 4 weeks to 12 weeks. However, patients took CDDP or nitrates with the same duration in the same study. Twenty-two RCTs reported the effective rate and the markedly effective rate in symptom improvement (17, 19–23, 26, 27, 29–42). Fifteen RCTs reported the effective rate and the markedly effective rate in ECG improvement (17, 22, 24, 26, 27, 29, 31–34, 36–39, 42). Twenty-three RCTs reported the adverse drug reactions (15–26, 28, 30, 32, 33, 35, 38–43).

**Table 1:** Characteristics of included studies.

Author and Publication year	Sample size (E)	Sample size (C)	Intervention (E)	Intervention (C)	Dosage of CDDP	Duration of CDDP	Dosage of ID or IN	Duration of ID or IN	Outcomes
Liu 1997	62	30	CDDP	ID	810 mg/day	6 weeks	30 mg/day	6 weeks	AR
Zhou 1998	120	60	CDDP	IN	810 mg/day	2 months	30 mg/day	2 months	ERSI,MERSI,EREI, MEREI, AR
Ding 1999	52	50	CDDP	ID	810 mg/day	2 months	30 mg/day	2 months	ERSI,MERSI, AR
Zhang 1999	69	64	CDDP	ID	810 mg/day	30 days	30 mg/day	30 days	ERSI,MERSI, AR
Gao 2000	60	60	CDDP	ID	810 mg/day	8 weeks	30 mg/day	8 weeks	ERSI,MERSI,EREI, MEREI,AR
Tan 2001	40	40	CDDP	ID	810 mg/day	4 weeks	30 mg/day	4 weeks	ERSI,MERSI,EREI, MEREI
Lu 2001	60	60	CDDP	ID	810 mg/day	8 weeks	30 mg/day	8 weeks	ERSI,MERSI,EREI, MEREI
Zhu 2001	80	80	CDDP	ID	810 mg/day	8 weeks	30 mg/day	8 weeks	ERSI,MERSI,EREI, MEREI
Yang 2001	43	40	CDDP	ID	810 mg/day	8 weeks	30 mg/day	8 weeks	ERSI,MERSI,EREI, MEREI, AR
Shi 2001	102	51	CDDP	ID	810 mg/day	4 weeks	30 mg/day	4 weeks	ERSI,MERSI, AR
Dong 2001	30	30	CDDP	ID	810 mg/day	4 weeks	30 mg/day	4 weeks	ERSI,MERSI,EREI, MEREI,AR
Xue 2001	102	90	CDDP	ID	810 mg/day	45 days	30 mg/day	45 days	ERSI,MERSI,EREI, MEREI,AR
Guo 2001	25	25	CDDP	ID	810 mg/day	8 weeks	30 mg/day	8 weeks	ERSI,MERSI,EREI, MEREI
Deng 2002	56	30	CDDP	ID	810 mg/day	8 weeks	30 mg/day	8 weeks	ERSI,MERSI, AR
Yao 2002a	50	50	CDDP	ID	810 mg/day	8 weeks	30 mg/day	8 weeks	ERSI,MERSI,EREI, MEREI
Yao 2002b	50	50	CDDP	ID	810 mg/day	4 weeks	30 mg/day	4 weeks	AR
Wang 2003	52	50	CDDP	ID	810 mg/day	2 months	30 mg/day	2 months	ERSI,MERSI,EREI, MEREI, AR
Chu 2003	35	32	CDDP	ID	810 mg/day	60 days	30 mg/day	60 days	ERSI,MERSI,EREI, MEREI
Huang 2004	40	38	CDDP	ID	810 mg/day	2 months	30 mg/day	2 months	AR
Li 2005	70	60	CDDP	IN	810 mg/day	6 weeks	30 mg/day	6 weeks	EREI,MEREI, AR
Li 2006	56	50	CDDP	IN	810 mg/day	6 weeks	30 mg/day	6 weeks	ERSI,MERSI, AR
Chi 2007	120	114	CDDP	ID	810 mg/day	4 weeks	30 mg/day	4 weeks	ERSI,MERSI,EREI, MEREI,AR
Yue 2008	55	53	CDDP	IN	810 mg/day	1 month	30 mg/day	1 month	ERSI,MERSI, AR
Pan 2008	50	30	CDDP	ID	810 mg/day	6 weeks	30 mg/day	6 weeks	ERSI,MERSI, AR
Luo 2008	54	52	CDDP	IN	810 mg/day	1 month	30 mg/day	1 month	ERSI,MERSI, AR
Wang 2009	29	28	CDDP	ID	810 mg/day	6 weeks	30 mg/day	6 weeks	AR
Zhang 2009	30	30	CDDP	ID	810 mg/day	8 weeks	30 mg/day	8 weeks	ERSI,MERSI, EREI, MEREI,AR
Gong 2013	60	60	CDDP	IN	810 mg/day	12 weeks	30 mg/day	12 weeks	AR
Shao 2013	66	60	CDDP	IN	810 mg/day	1 month	30 mg/day	1 month	AR

E, Experimental group; C, Control group; CDDP, compound danshen dripping pills; ID, isosorbide dinitrate; IN, isosorbide nitrate; ERSI, Effective rate in symptom improvement; MERSI, Markedly effective rate in symptom improvement; EREI, Effective rate in ECG improvement; MEREI, Markedly effective rate in ECG improvement; AR, Adverse reaction.

### 3.3. Assessment of risk of bias

**Figure 2:**
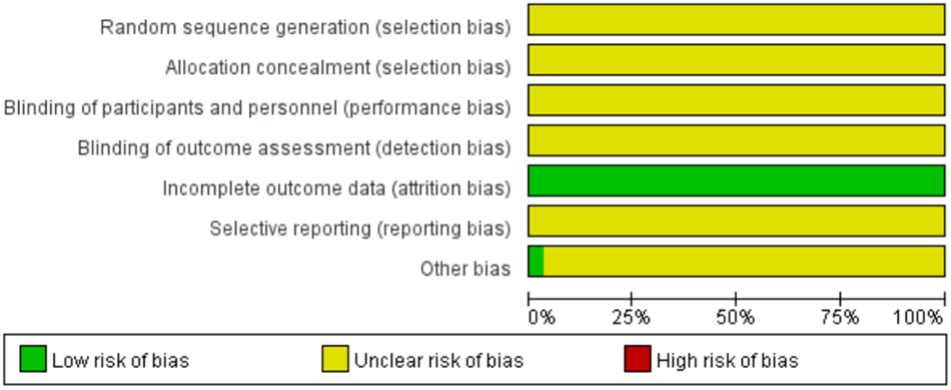
Risk of bias graph.

**Figure 3:**
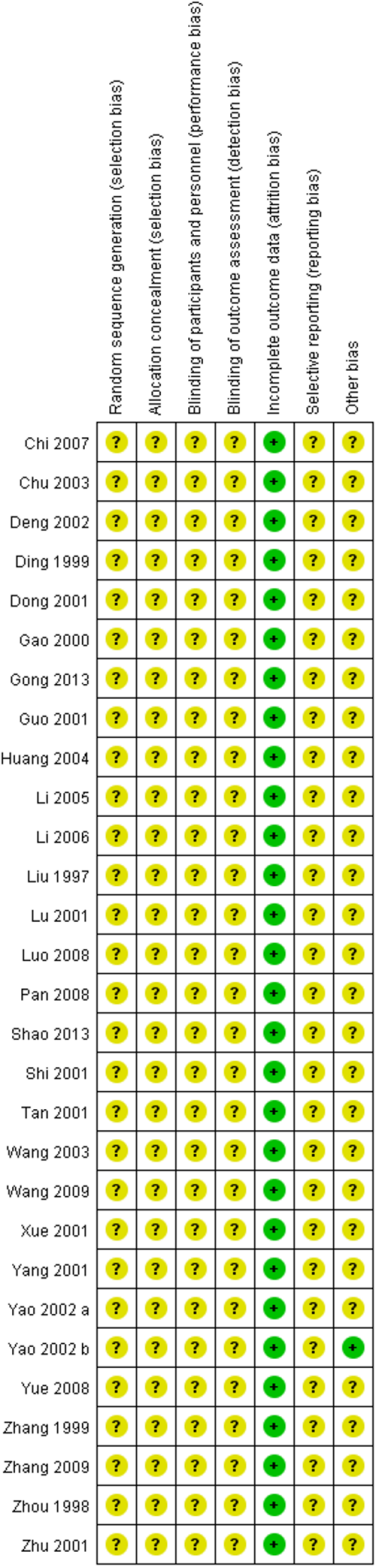
Risk of bias summary.

Figures 2, 3 show the results of risk of bias assessment. The risk of selection bias was graded as unclear in all of the included studies because no specific random sequence generation and allocation concealment methods were reported. The risk of performance bias was also classified as unclear in all of the included studies because no blinding of participants, personnel or outcome assessment was clearly reported. The risk of attrition bias was low in all of the included studies due to the complete outcome data. The reporting bias and other bias were categorized as unclear risk because of the insufficient information.

### 3.4. Symptom improvement

#### 3.4.1. Effective rate in symptom improvement

**Figure 4:**
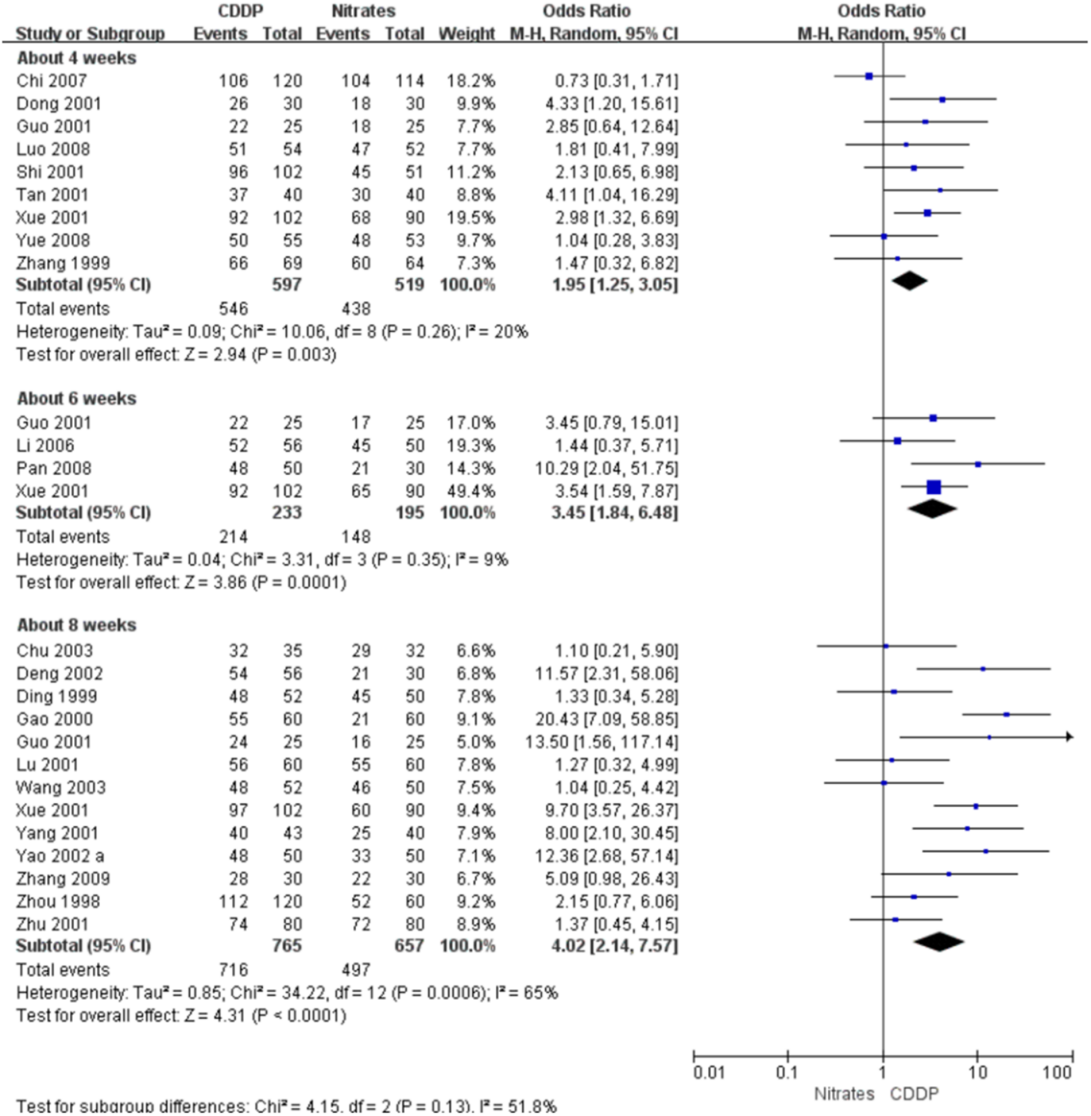
Meta-analyses on effective rate in symptom improvement.

Twenty-two RCTs reported the effective rate in symptom improvement. The meta-analyses with the random-effect model indicated that CDDP could significantly increase the effective rate in symptom improvement compared with nitrates (Pooled 9 RCTs, OR = 1.95, 95% CI: 1.25–3.05, *P* = 0.003, duration of about 4 weeks; Pooled 4 RCTs, OR = 3.45, 95% CI: 1.84–6.48, *P* = 0.0001, duration of about 6 weeks; Pooled 13 RCTs, OR = 4.02, 95% CI: 2.14–7.57, *P *< 0.0001, duration of about 8 weeks; Figure 4). The results of the meta-analyses with fixed-effect model were similar with above results in Table 2. In addition, one RCT (29) reported that the effective rate in symptom improvement in the CDDP group was higher than that in the nitrates group (OR = 1.5, 95% CI: 0.68–3.31, *P* = 0.32) after 5 weeks of CDDP treatment.

**Table 2:** Meta-analyses with fixed-effect model.

Outcomes	Duration of CDDP treatment	Number of included studies	OR	95% CI		*P* value
				Lower limit	Upper limit	
Effective rate in symptom improvement	4 weeks	8	1.95	1.32	2.87	0.0007
	6 weeks	4	3.52	1.99	6.24	<0.0001
	8 weeks	13	4.57	3.26	6.4	<0.00001
Markedly effective rate in symptom improvement	4 weeks (standard 1)	3	1.26	0.75	2.1	0.38
	4 weeks (standard 2)	6	1.54	1.15	2.05	0.003
	6 weeks (standard 1)	2	1.56	0.86	2.81	0.14
	6 weeks (standard 2)	2	1.43	0.77	2.65	0.26
	8 weeks (standard 1)	9	2.73	2.04	3.64	<0.00001
	8 weeks (standard 2)	4	2.36	1.59	3.5	<0.0001
Effective rate in ECG improvement	4 weeks	5	1.49	1.07	2.08	0.02
	6 weeks	3	2.47	1.6	3.82	<0.0001
	8 weeks	11	3.44	2.7	4.39	<0.00001
Markedly effective rate in ECG improvement	4 weeks	5	1.62	1.07	2.47	0.02
	6 weeks	3	2.34	1.4	3.9	0.001
	8 weeks	11	2.37	1.76	3.18	<0.00001
Adverse drug reactions	—	23	0.12	0.09	0.17	<0.00001

#### 3.4.2. Markedly effective rate in symptom improvement

**Figure 5:**
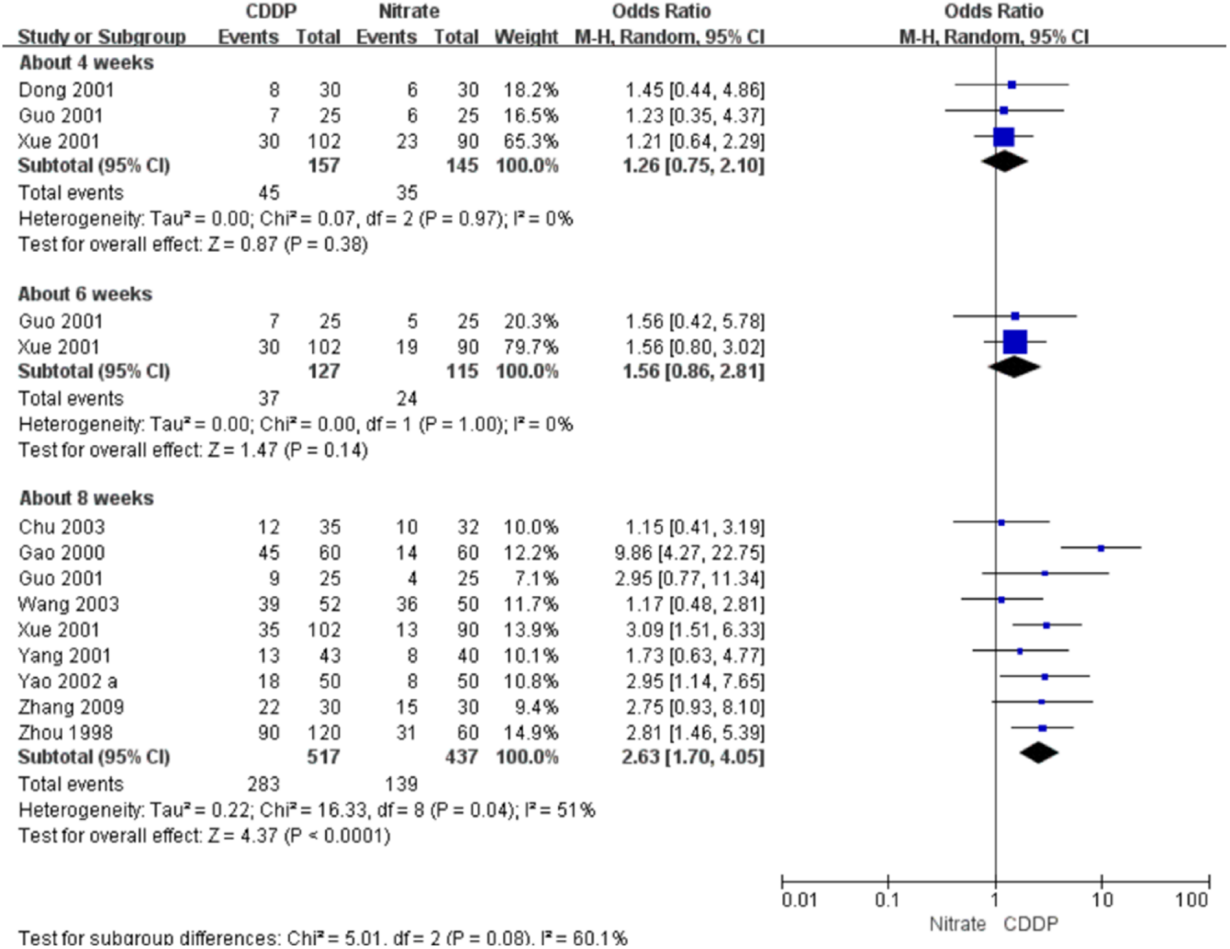
Meta-analyses on markedly effective rate in symptom improvement (standard 1).

**Figure 6:**
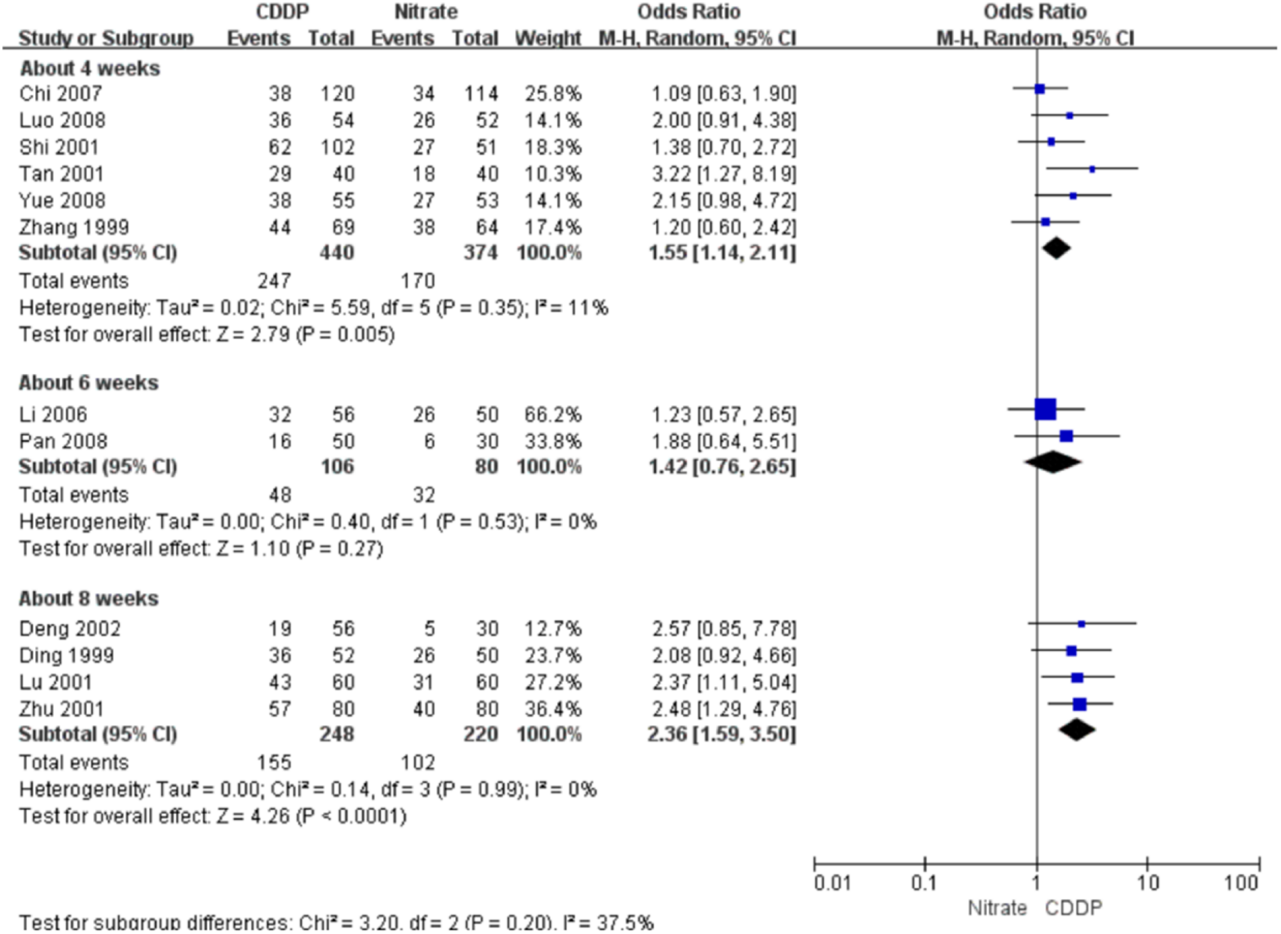
Meta-analyses on markedly effective rate in symptom improvement (standard 2).

Twenty-two RCTs reported the markedly effective rate in symptom improvement. When the markedly effective in symptom improvement was defined as “symptoms associated with SAP were disappeared, the frequency of angina attacks or nitroglycerin consumption was reduced by more than 80% after treatment” (standard 1), the meta-analyses with the random-effect model indicated that CDDP compared with nitrates could increase the markedly effective rate in symptom improvement with no statistical significance at 4 and 6 weeks after treatment (Pooled 3 RCTs, OR = 1.26, 95% CI: 0.75–2.10, *P* = 0.38, duration of about 4 weeks; Pooled 2 RCTs, OR = 1.56, 95% CI: 0.86–2.81, *P* = 0.14, duration of about 6 weeks; Figure 5) and with the statistical significance at 8 weeks after treatment (Pooled 9 RCTs, OR = 2.63, 95% CI: 1.70–4.05, *P* < 0.0001, duration of about 8 weeks; Figure 5). When the markedly effective in symptom improvement was defined as “symptoms associated with SAP were disappeared, the frequency of angina attacks or nitroglycerin consumption was reduced by more than 90% after treatment” (standard 2), the meta-analyses with the random-effect model indicated that CDDP compared with nitrates could increase the markedly effective rate in symptom improvement with no statistical significance at 6 weeks after treatment (Pooled 2 RCTs, OR = 1.42, 95% CI: 0.76–2.65, *P* = 0.27, duration of about 6 weeks; Figure 6) and with the statistical significance at 4 and 8 weeks after treatment (Pooled 6 RCTs, OR = 1.55, 95% CI: 1.14–2.11, *P* = 0.005, duration of about 4 weeks; Pooled 4 RCTs, OR = 2.36, 95% CI: 1.59–3.50, *P* < 0.0001, duration of about 8 weeks; Figure 6). The results of the meta-analyses with fixed-effect model were similar with above results in Table 2.

### 3.5. ECG improvement

#### 3.5.1. Effective rate in ECG improvement

**Figure 7:**
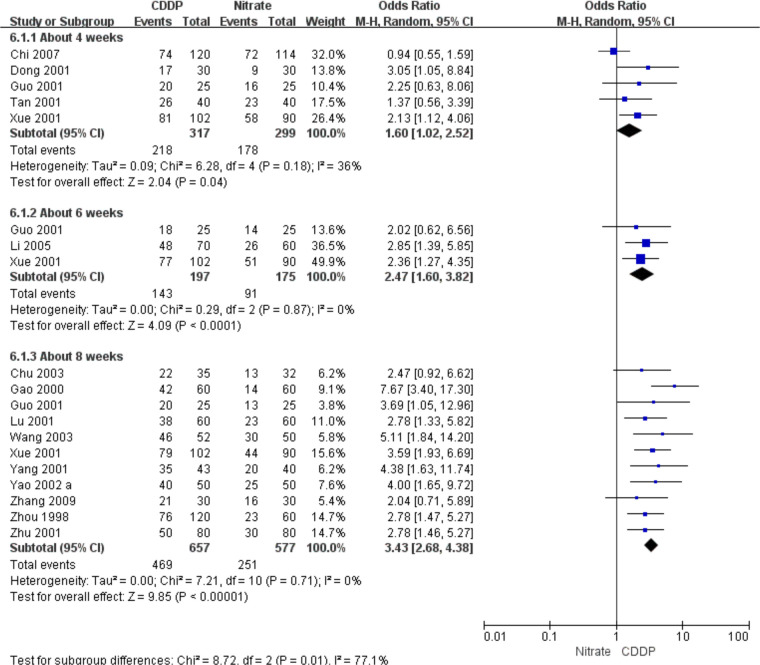
Meta-analyses on effective rate in ECG improvement.

Fifteen RCTs reported the effective rate in ECG improvement. The meta-analyses with the random-effect model indicated that CDDP could significantly increase the effective rate in ECG improvement compared with nitrates (Pooled 5 RCTs, OR = 1.60, 95% CI: 1.02–2.52, *P* = 0.04, duration of about 4 weeks; Pooled 3 RCTs, OR = 2.47, 95% CI: 1.60–3.82, *P* < 0.0001, duration of about 6 weeks; Pooled 11 RCTs, OR = 3.43, 95% CI: 2.68–4.38, *P *< 0.00001, duration of about 8 weeks; Figure 7). The results of the meta-analyses with fixed-effect model were similar with above results in Table 2.

#### 3.5.2. Markedly effective rate in ECG improvement

**Figure 8:**
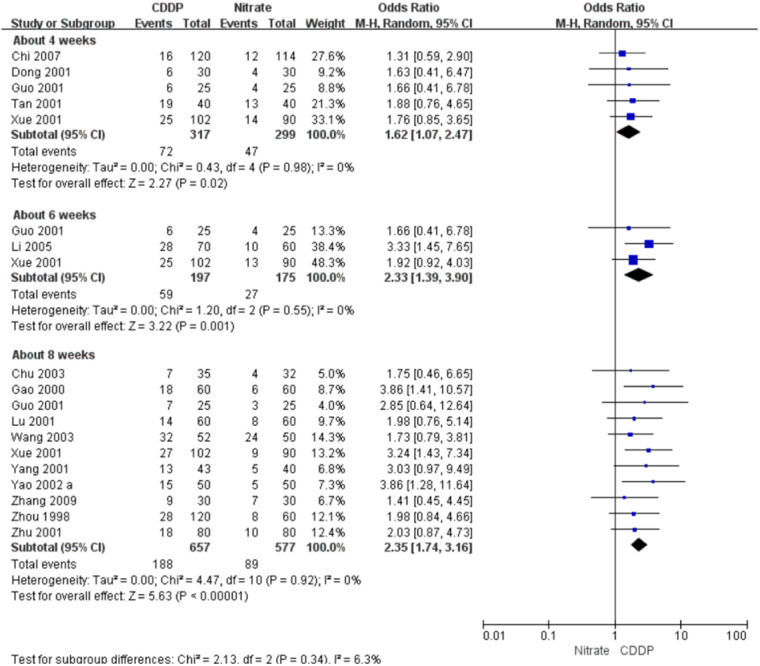
Meta-analyses on markedly effective rate in ECG improvement.

Fifteen RCTs reported the markedly effective rate in ECG improvement. The meta-analyses with the random-effect model indicated that CDDP could significantly increase the markedly effective rate in ECG improvement compared with nitrates (Pooled 5 RCTs, OR = 1.62, 95% CI: 1.07–2.47, *P* = 0.02, duration of about 4 weeks; Pooled 3 RCTs, OR = 2.33, 95% CI: 1.39–3.90, *P* = 0.001, duration of about 6 weeks; Pooled 11 RCTs, OR = 2.35, 95% CI: 1.74–3.16, *P* < 0.00001, duration of about 8 weeks; Figure 8). The results of the meta-analyses with fixed-effect model were similar with above results in Table 2.

### 3.6. Adverse drug reactions

**Figure 9:**
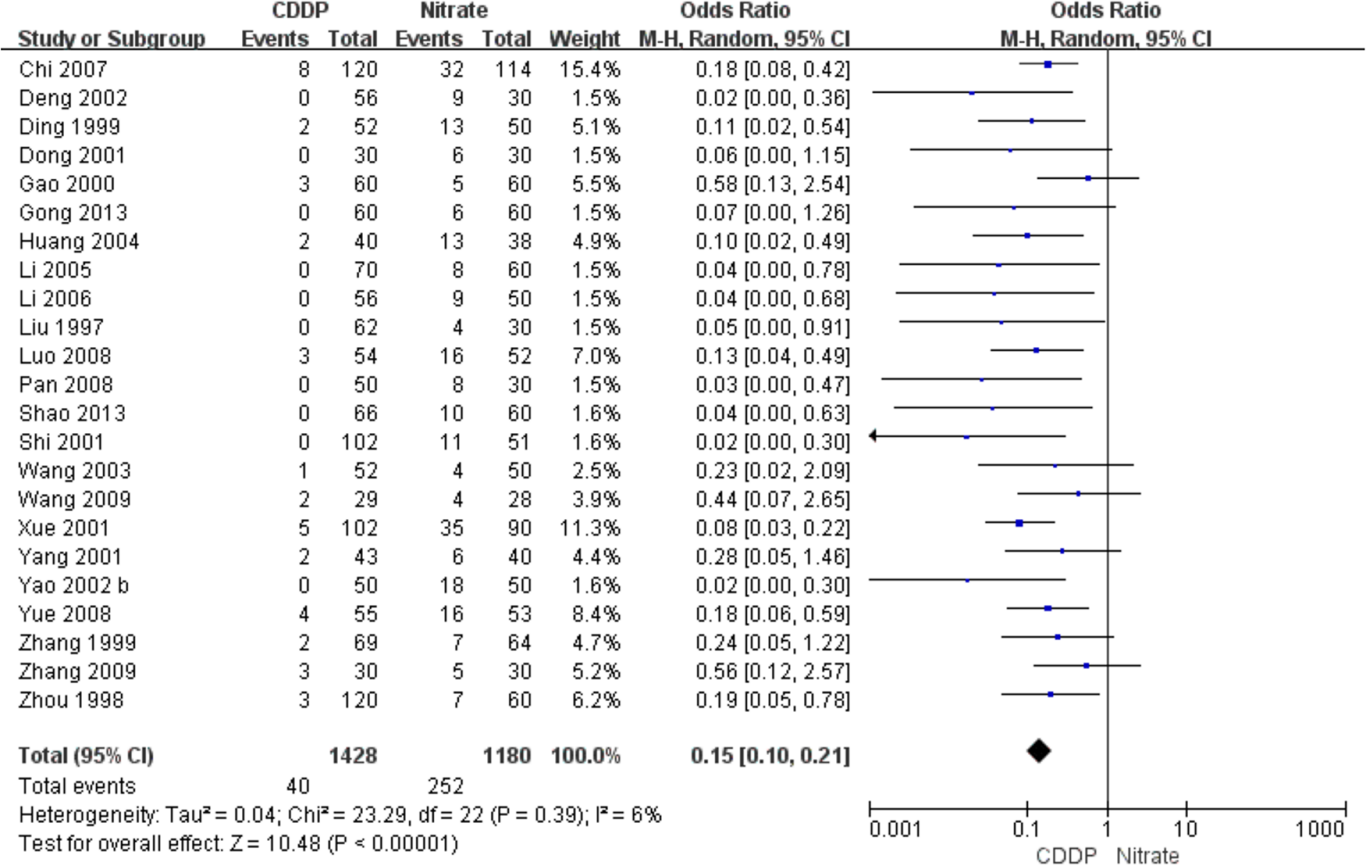
Meta-analysis on the adverse drug reactions.

Twenty-three RCTs reported the adverse drug reactions. The pooled result showed that the incidence of adverse drug reactions in the CDDP group was lower than that in the nitrates group (Pooled 23 RCTs, OR = 0.15, 95% CI: 0.1–0.21, *P* < 0.00001, Figure 9). Specifically, adverse drug reactions included stomach discomfort in the CDDP group. In the nitrate group, adverse drug reactions included headache, dizziness, facial burning, nausea, etc.

### 3.7. Publication bias

**Figure 10:**
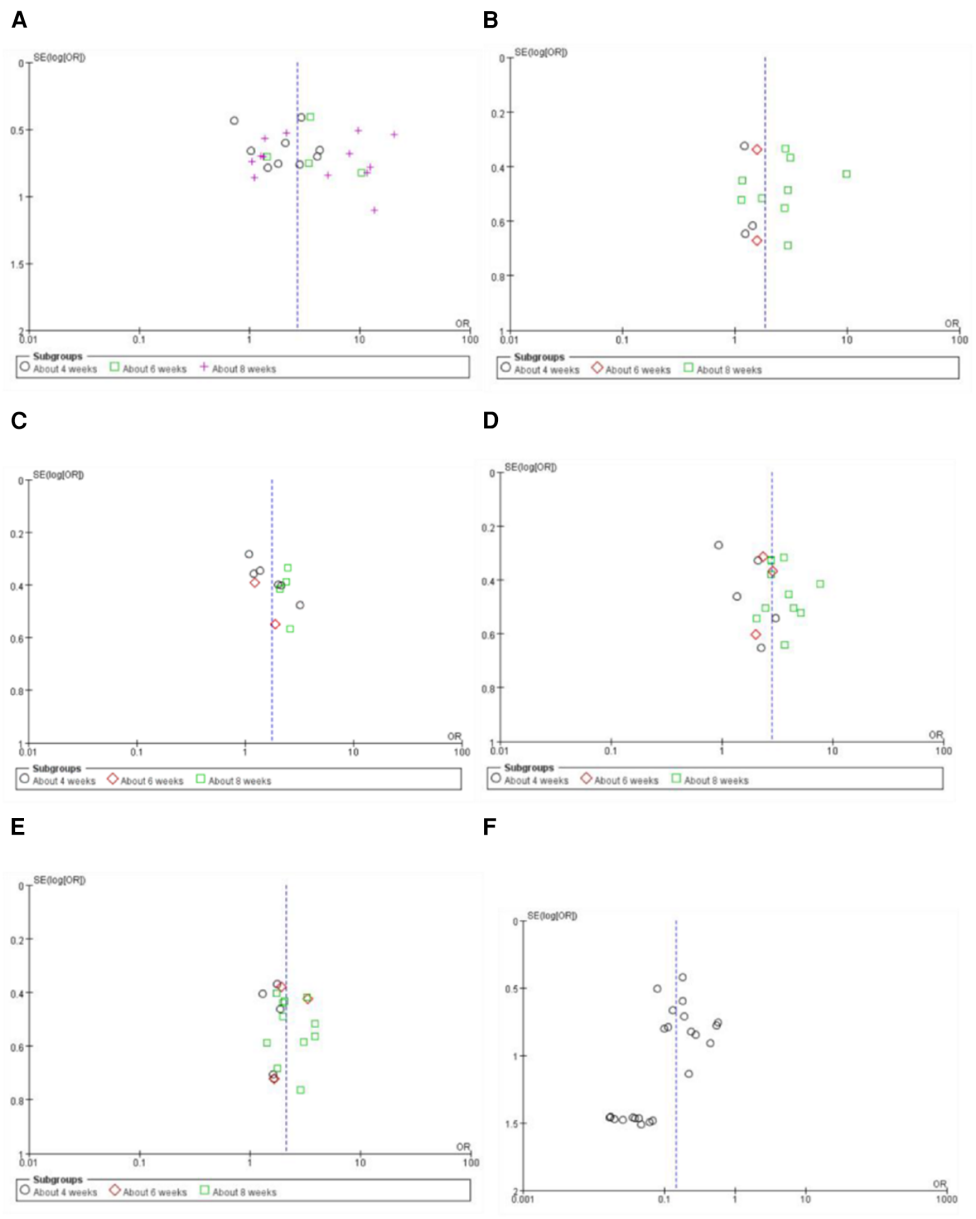
Funnel plot of the meta-analyses on effective rate in symptom improvement (**A**), markedly effective rate in symptom improvement (standard 1) (**B**), markedly effective rate in symptom improvement (standard 2) (**C**), effective rate in ECG improvement (**D**), markedly effective rate in ECG improvement (**E**), and the adverse drug reaction (**F**).

Funnel plots of the meta-analyses are presented in Figure 10. Publication bias may be existed because of the asymmetry in some funnel plots. It may be associated with small sample size, poor methodological quality, etc.

### 3.8. Grade of evidence

The grade of evidence for each outcome was presented in Table 3. The levels of the evidence for the effective rate and the markedly effective rate in symptom improvement ranged from very low to low. The levels of the evidence for the effective rate and markedly effective rate in ECG improvement were classified as low.

**Table 3:** GRADE quality of evidence summary table.

Outcomes	Illustrative Comparative Risks (95% CI)		OR (95% CI)	No. of Participants (Studies)	Quality of the Evidence
	Assumed Risk (Nitrates)	Corresponding Risk (CDDP)			
Effective rate in symptom improvement (CDDP for about 4 weeks)	844 per 1,000	913 per 1,000 (871–943)	1.95 (1.25–3.05)	1,116 (9 studies)	⊕⊕⊖⊖ Low*
Effective rate in symptom improvement (CDD for about 6 weeks)	759 per 1,000	916 per 1,000 (853–953)	3.45 (1.84–6.48)	428 (4 studies)	⊕⊕⊖⊖ Low*
Effective rate in symptom improvement (CDD for about 8 weeks)	756 per 1,000	926 per 1,000 (869–959)	4.02 (2.14–7.57)	1,422 (13 studies)	⊕⊖⊖⊖ Very low*^,^^†^
Markedly effective rate in symptom improvement (Srandard 1, CDDP for about 4 weeks)	241 per 1,000	286 per 1,000 (193–401)	1.26 (0.75–2.1)	302 (3 studies)	⊕⊕⊖⊖ Low*
Markedly effective rate in symptom improvement (Srandard 2, CDDP for about 4 weeks)	455 per 1,000	564 per 1,000 (487–637)	1.55 (1.14–2.11)	814 (6 studies)	⊕⊕⊖⊖ Low*
Markedly effective rate in symptom improvement (Srandard 1, CDDP for about 6 weeks)	209 per 1,000	291 per 1,000 (185–426)	1.56 (0.86–2.81)	242 (2 studies)	⊕⊕⊖⊖ Low*
Markedly effective rate in symptom improvement (Srandard 2, CDDP for about 6 weeks)	400 per 1,000	486 per 1,000 (336–639)	1.42 (0.76–2.65)	186 (2 studies)	⊕⊕⊖⊖ Low*
Markedly effective rate in symptom improvement (Srandard 1, CDDP for about 8 weeks)	318 per 1,000	551 per 1,000 (442–654)	2.63 (1.7–4.05)	954 (9 studies)	⊕⊖⊖⊖ Very low*^,^^†^
Markedly effective rate in symptom improvement (Srandard 2, CDDP for about 8 weeks)	464 per 1,000	671 per 1,000 (579–752)	2.36 (1.59–3.5)	468 (4 studies)	⊕⊕⊖⊖ Low*
Effective rate in ECG improvement (CDDP for about 4 weeks)	595 per 1,000	702 per 1,000 (600–788)	1.60 (1.02–2.52)	616 (5 studies)	⊕⊕⊖⊖ Low*
Effective rate in ECG improvement (CDDP for about 6 weeks)	520 per 1,000	728 per 1,000 (634–805)	2.47 (1.60–3.82)	372 (3 studies)	⊕⊕⊖⊖ Low*
Effective rate in ECG improvement (CDDP for about 8 weeks)	435 per 1,000	725 per 1,000 (674–771)	3.43 (2.68–4.38)	1,234 (11 studies)	⊕⊕⊖⊖ Low*
Markedly effective rate in ECG improvement (CDDP for about 4 weeks)	157 per 1,000	232 per 1,000 (166–315)	1.62 (1.07–2.47)	616 (5 studies)	⊕⊕⊖⊖ Low*
Markedly effective rate in ECG improvement (CDDP for about 6 weeks)	154 per 1,000	298 per 1,000 (202–416)	2.33 (1.39–3.90)	372 (3 studies)	⊕⊕⊖⊖ Low*
Markedly effective rate in ECG improvement (CDDP for about 8 weeks)	154 per 1,000	300 per 1,000 (214–366)	2.35 (1.74–3.16)	1,234 (11 studies)	⊕⊕⊖⊖ Low*
Adverse drug reactions	214 per 1,000	39 per 1,000 (26–54)	0.15 (0.1–0.21)	2,608 (23 studies)	⊕⊕⊖⊖ Low*

CDDP, Compound danshen dropping pills; CI, Confidence interval; OR, Odds ratio.

^*^ Unclear risk of bias due to limitations of randomization, allocation concealment and blinding.

^†^ I2 was more than 50%.

## 4. Discussion

Nitrates have been widely used in the management of angina in the clinical practice (44). However, the long-term use of nitrates may be limited because of the tolerance to nitrates (44). It may be caused by multiple factors, such as a burst of oxygen free radicals and vascular peroxynitrite formation (45–47). Many efforts are made to reduce the tolerance to nitrates, such as the intermittent nitrate therapy and long-acting nitrates (48). However, intermittent nitrate therapy may bring about the rebound ischemia (49), and long-acting nitrates may be associated with the endothelial dysfunction (48). At present, novel nitrate drugs are still under development (50, 51). Some side effects associated with nitrates for treating angina have been reported, such as headache, dizziness, and hypotension (52). Therefore, alternative therapies for angina are still needed.

CDDP has been approved for treating angina associated with coronary heart disease by the National Medical Products Administration in China for over twenty years. Many RCTs comparing CDDP with nitrates for treating angina has been published in recent years. A systematic review in 2015 suggested that CDDP were more effective than isosorbide dinitrate in improving the symptoms and ECG in patients with angina (53). However, some limitations on that study can't be ignored. For example, only isosorbide dinitrate as one type of nitrates was considered as the control intervention. The safety was not assessed and the subgroup analysis based on stable and unstable angina was not conducted. Another systematic review in 2021 did not conduct subgroup analyses based on the duration of the drugs, and did not report adverse drug reactions (54). In this study, an updated systematic review with the strict eligibility criteria was conducted to critically evaluate the efficacy and safety of CDDP vs. nitrates for treating SAP. The meta-analyses were conducted based on the duration of CDDP. The pooled results showed that the effective rate in symptom improvement and ECG improvement were significantly increased at about 4, 6 or 8 weeks after CDDP treatment compared to nitrates. The incidence of adverse reactions in the CDDP group was lower than that in the nitrates group. It means that CDDP with the duration of at least 4 weeks has the relative advantages in relieving symptoms associated with SAP, reducing the frequency of angina attacks or nitroglycerin consumption, and has relatively fewer adverse effects compared with nitrates. It provides a new insight into the management of SAP. CDDP with the duration of at least 4 weeks may be considered as an alternative to nitrates for treating SAP.

Several limitations should be taken into consideration when interpreting above results. Firstly, the effect size may be overestimated or underestimated due to the small sample size (less than 100) in most of included studies. Secondly, the risks of selection bias, performance bias, reporting bias and other bias for most of included studies were graded as unclear because of insufficient information. Thirdly, the mechanisms of CDDP for treating SAP aren't fully understood due to the lack of pharmacological evidence.

## 5. Conclusion

The present study suggests that CDDP with the duration of at least 4 weeks can be considered as an alternative to nitrates for treating SAP. However, more high-quality RCTs are still needed to confirm these findings.

## Data Availability

The original contributions presented in the study are included in the article/**Supplementary Material**, further inquiries can be directed to the corresponding authors.
