# Supplementary material for: Compound danshen dripping pills vs. nitrates for stable angina pectoris: a systematic review and meta-analysis
Source: Front Cardiovasc Med. 2023 May 22;10:1168730. doi: 10.3389/fcvm.2023.1168730 (PMC10240178; doi:10.3389/fcvm.2023.1168730)
Supplement: Supplementary file 1 [file Table1.docx]

Detailed search strategies:

Pubmed:

#1 "Fufang danshen diwan"[Title/Abstract] OR "Compound Danshen dropping pills"[Title/Abstract] OR T89[Title/Abstract] OR "Salvia miltiorrhiza"[Title/Abstract] OR Danshen[Title/Abstract]

#2 Nitrates[Title/Abstract] OR Nitroglycerin[Title/Abstract] OR NTG[Title/Abstract] OR Trinitrin[Title/Abstract] OR Anginine[Title/Abstract] OR "Glyceryl trinitrate" [Title/Abstract] OR "Isosorbide dinitrate" [Title/Abstract] OR "Isosorbide nitrate" [Title/Abstract] OR "Isosorbide mononitrate" [Title/Abstract] OR Isoket[Title/Abstract] OR GTN[Title/Abstract]

#3 "Angina pectoris"[Title/Abstract] OR Angina[Title/Abstract] OR stenocardia[Title/Abstract] OR "Angor pectoris"[Title/Abstract]

#4 ("randomized controlled trial"[Title/Abstract] OR "controlled clinical trial"[Title/Abstract] OR randomized[Title/Abstract] OR placebo[Title/Abstract] OR randomly[Title/Abstract] OR trial[Title/Abstract])

#5 #1 AND #2 AND #3 AND #4

Embase:

#1 ‘Fufang danshen diwan’:ab,ti,kw OR ‘Compound Danshen dropping pills’:ab,ti,kw OR T89:ab,ti,kw OR ‘Salvia miltiorrhiza’:ab,ti,kw OR Danshen:ab,ti,kw

#2 Nitrates:ab,ti,kw OR Nitroglycerin:ab,ti,kw OR NTG:ab,ti,kw OR Trinitrin:ab,ti,kw OR Anginine:ab,ti,kw OR ‘Glyceryl trinitrate’ :ab,ti,kw OR ‘Isosorbide dinitrate’ :ab,ti,kw OR ‘Isosorbide nitrate’ :ab,ti,kw OR ‘Isosorbide mononitrate’ :ab,ti,kw OR Isoket:ab,ti,kw OR GTN:ab,ti,kw

#3 ‘Angina pectoris’:ab,ti,kw OR Angina:ab,ti,kw OR stenocardia:ab,ti,kw OR ‘Angor pectoris’:ab,ti,kw

#4 (‘randomized controlled trial’:ab,ti,kw OR ‘controlled clinical trial’:ab,ti,kw OR randomized:ab,ti,kw OR placebo:ab,ti,kw OR randomly:ab,ti,kw OR trial:ab,ti,kw)

#5 #1 AND #2 AND #3 AND #4

Web of Science:

#1 TS=(Fufang danshen diwan) OR TS=(Compound Danshen dropping pills) OR TS=(T89) OR TS=(Salvia miltiorrhiza) OR TS=(Danshen)

#2 TS=(Nitrates) OR TS=(Nitroglycerin) OR TS=(NTG) OR TS=(Trinitrin) OR TS=(Anginine) OR TS=(Glyceryl trinitrate) OR TS=(Isosorbide dinitrate) OR TS=(Isosorbide nitrate ) OR TS=(Isosorbide mononitrate) OR TS=(Isoket) OR TS=(GTN)

#3 TS=(Angina pectoris) OR TS=(Angina) OR TS=(stenocardia) OR TS=(Angor pectoris)

#4 TS=(randomized controlled trial) OR TS=(controlled clinical trial) OR TS=(randomized) OR TS=(placebo) OR TS=(randomly) OR TS=(trial)

#5 #1 AND #2 AND #3 AND #4

Cochrane library:

#1 ‘Fufang danshen diwan’:ti,ab,kw OR ‘Compound Danshen dropping pills’:ti,ab,kw OR T89:ti,ab,kw OR ‘Salvia miltiorrhiza’:ti,ab,kw OR Danshen:ti,ab,kw

#2 Nitrates:ti,ab,kw OR Nitroglycerin:ti,ab,kw OR NTG:ti,ab,kw OR Trinitrin:ti,ab,kw OR Anginine:ti,ab,kw OR ‘Glyceryl trinitrate’:ti,ab,kw OR ‘Isosorbide dinitrate’:ti,ab,kw OR ‘Isosorbide nitrate’:ti,ab,kw OR ‘Isosorbide mononitrate’:ti,ab,kw OR Isoket:ti,ab,kw OR GTN:ti,ab,kw

#3 ‘Angina pectoris’:ti,ab,kw OR Angina:ti,ab,kw OR stenocardia:ti,ab,kw OR ‘Angor pectoris’:ti,ab,kw

#4 (‘randomized controlled trial’:ti,ab,kw OR ‘controlled clinical trial’:ti,ab,kw OR randomized:ti,ab,kw OR placebo:ti,ab,kw OR randomly:ti,ab,kw OR trial:ti,ab,kw)

#5 #1 AND #2 AND #3 AND #4

China National Knowledge Infrastructure (CNKI):

(SU=复方丹参滴丸 OR SU=复方丹参) AND (SU=硝酸盐 OR SU=硝酸甘油 OR SU=硝酸异山梨酯OR SU=单硝酸异山梨酯 OR SU=消心痛) AND (SU=试验 OR SU=观察 OR SU=随机 OR SU=对照)

Wanfang Digital Periodicals (WANFANG):

(主题词:复方丹参滴丸 OR 主题词:复方丹参) AND (主题词:硝酸盐 OR 主题词:硝酸甘油 OR 主题词:硝酸异山梨酯OR 主题词:单硝酸异山梨酯 OR 主题词:消心痛) AND (主题词:试验 OR 主题词:观察 OR 主题词:随机 OR 主题词:对照)

Chinese Science and Technology Periodicals (VIP) database:

U=(复方丹参滴丸 OR 复方丹参 OR 丹参) AND U=(硝酸盐 OR 硝酸甘油 OR 硝酸异山梨酯OR 单硝酸异山梨酯 OR 消心痛) AND U=(试验 OR 观察 OR 随机 OR 对照)
